# IS3 profiling identifies the enterohaemorrhagic *Escherichia coli *O-island 62 in a distinct enteroaggregative *E. coli *lineage

**DOI:** 10.1186/1757-4749-3-4

**Published:** 2011-03-30

**Authors:** Iruka N Okeke, Louissa R Macfarlane-Smith, Jonathan N Fletcher, Anna M Snelling

**Affiliations:** 1 Department of Biology, Haverford College, 370 Lancaster Avenue, Haverford, PA 19041, USA; 2 Division of Biomedical Sciences, University of Bradford, Richmond Road, Bradford, West Yorkshire, BD7 1DP, UK; 3 Bradford Infection Group, University of Bradford, Richmond Road, Bradford, West Yorkshire, BD7 1DP, UK; 4 Department of Microbiology, Leeds General Infirmary, Old Medical School, Thoresby Place, Leeds, LS1 3EX, UK

## Abstract

**Background:**

Enteroaggregative *Escherichia coli *(EAEC) are important diarrhoeal pathogens that are defined by a HEp-2 adherence assay performed in specialist laboratories. Multilocus sequence typing (MLST) has revealed that aggregative adherence is convergent, providing an explanation for why not all EAEC hybridize with the plasmid-derived probe for this category, designated CVD432. Some EAEC lineages are globally disseminated or more closely associated with disease.

**Results:**

To identify genetic loci conserved within significant EAEC lineages, but absent from non-EAEC, IS3-based PCR profiles were generated for 22 well-characterised EAEC strains. Six bands that were conserved among, or missing from, specific EAEC lineages were cloned and sequenced. One band corresponded to the *aggR *gene, a plasmid-encoded regulator that has been used as a diagnostic target but predominantly detects EAEC bearing the plasmid already marked by CVD432. The sequence from a second band was homologous to an open-reading frame within the cryptic enterohaemorrhagic *E. coli *(EHEC) O157 genomic island, designated O-island 62. Screening of an additional 46 EAEC strains revealed that the EHEC O-island 62 was only present in those EAEC strains belonging to the ECOR phylogenetic group D, largely comprised of sequence type (ST) complexes 31, 38 and 394.

**Conclusions:**

The EAEC 042 gene orf1600, which lies within the EAEC equivalent of O-island 62 island, can be used as a marker for EAEC strains belonging to the ECOR phylogenetic group D. The discovery of EHEC O-island 62 in EAEC validates the genetic profiling approach for identifying conserved loci among phylogenetically related strains.

## Background

Enteroaggregative *Escherichia coli *(EAEC) were originally associated with persistent diarrhoea in developing countries but are now known to cause both acute and persistent diarrhoea worldwide [1]. EAEC strains all demonstrate a characteristic aggregative adherence to human epithelial cells *in vivo *or in culture. There are no other phenotypic or genotypic properties known to be shared by all EAEC strains, and the contribution of potential EAEC virulence factors to human disease is yet to be assessed. Volunteer studies and outbreaks have unequivocally demonstrated that at least some EAEC strains are pathogens [2-5]. However, epidemiological studies have always recovered EAEC from healthy people as well as individuals with diarrhoea. Although host factors are one reason for this observation [6,7], it is almost certain that not all EAEC strains are pathogenic.

The Gold Standard for EAEC detection is the HEp-2 adherence assay. As this assay can only be performed in specialised research and reference laboratories, most epidemiological studies employ a DNA probe, CVD432 to detect EAEC. This is an empirically identified fragment derived from the aggregative plasmid of Chilean isolate 17-2 [8]. It is now known to be part of an operon encoding an export system for the enteroaggregative secreted anti-aggregative protein, Aap, also known as dispersin [9]. The CVD432 probe was originally shown to have a sensitivity of 89% and a specificity of 99% [8]. However, more recent and inclusive studies have shown that although it maintains specificity, the sensitivity of the probe varies from under 20% to over 80% [10]. As most epidemiological studies have used this probe alone to identify EAEC, their importance in diarrhoea is currently underestimated and the true, overall sensitivity of the CVD432 probe is unknown. Moreover, plasmids that bear this locus do not have a conserved backbone [11,12].

Genetic studies are needed to identify alternatives or supplements to the currently available probe. Furthermore, upon completion of the sequence analysis of the genome of the CVD432-positive EAEC strain 042 [13], emphasized the need to determine which genes are present in other EAEC strains. Multilocus sequence typing of 150 EAEC strains recently revealed that EAEC strains are distributed throughout the *E. coli *phylogeny but that closely related EAEC strains did share some known virulence genes. For example, most EAEC strains belonging to the ECOR group D (principally ST complex 31, 38 and 394 strains) carry long polar fimbriae genes, a chromosomal antimicrobial resistance island, the heat-resistant agglutinin gene and the pathogenicity island-encoded *fepC *gene [12]. Additionally, epidemiological association of EAEC with disease varies with different lineages with ST complexes 38 and 394 (ECOR group D) and 10 (ECOR group A) less commonly recovered from healthy individuals in Nigeria. Thus, the aggregative adherence phenotype emerged independently in multiple EAEC lineages and the EAEC category as defined by adherence pattern alone is likely to be comprised of strains that have different pathogenic mechanisms [12].

In this study, we attempted to identify other genetic loci that are common to strains belonging to globally disseminated EAEC lineages. We used IS-3 profiling, a PCR-profiling method that takes advantage of the fact that *E. coli *strains typically have multiple copies of insertion-sequence 3 at different locations in the genome [14-16]. The profiling is performed at low stringency so that loci distant from IS3 elements may also be amplified. Our objective was to identify loci that, unlike previously described conserved genes, are not necessarily plasmid borne, and are uncommon in non-EAEC. Such loci could be candidate targets for diagnostic tests.

## Results

### IS3-based PCR profiling confirms EAEC heterogeneity and identifies a locus present in ST31- and ST394-complex EAEC strains

**Figure 1:**
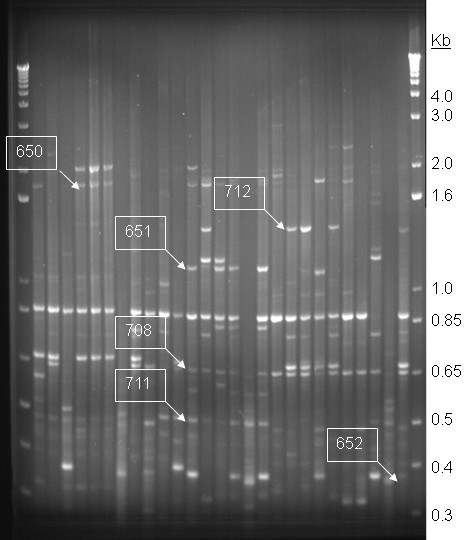
**Typical IS3 profiling gel**. Lanes 1 and 29: 1 Kb ladder plus (Invitrogen); Lanes 2-12: EAEC strains AA 60A, NA H191-1, AA H232-1, AA 17-2, AA 253-1, AA 6-1, AA DS65-R2, AA501-1, AA H223-1, DA WC212-11 and AA DS67-R2; Lanes 13-25: AA H38-1, AA 042, AA 144-1, AA 44-1, AA H145-1, AA 309-1, AA 103-1, DA H92-1, AA 435-1, AA 199-1, AA H194-2, AA 278-1 and AA 239-1; Lanes 26-28: Control strains EHEC O157 EDL933, *Shigella flexneri *2a 2425T and *E. coli *K-12 MG1655. Boxed numbers indicate bands described in Table 1.

IS3-based PCR profiling is less discriminatory than pulsed-field gel electrophoresis and generates much smaller band sizes, which made it suitable for isolating conserved bands for characterisation [16]. Since we observed 20 non-identical profiles among 22 EAEC reference strains belonging to 15 STs, IS3-profiling was more discriminatory than MLST. However, there were bands common across multiple related STs, allowing us to identify loci that might be conserved among them. The diversity of profiles seen in this study adds to existing information that points to considerable heterogeneity among EAEC. The data shows that there is also genetic diversity within common EAEC STs, such as ST10, ST34 and ST31, but there are some profile similarities within these groups (Figure 1).

There were no bands of identical size that amplified from all EAEC but were absent in non-EAEC controls. Nine band-sizes were of interest because they were either present or absent in most EAEC strains or specific STs/ST complexes. Three of these bands did not amplify during more than one screening and were therefore not examined further. We were able to reproducibly amplify and clone six bands, which were end-sequenced from plasmid clones (Table 1). Four bands contained DNA that originated from housekeeping genes, which gene-specific PCR demonstrated were also present in strains that lacked the band (data not shown). Therefore the banding pattern is likely to be due to absence of a proximal IS3 element or other complementary DNA for priming. One band represented a region adjacent to the *aggR *gene, encoding the aggregative adherence regulator [17]. IS3 elements are now known to be frequently found on large virulence plasmids, particularly EAEC plasmids, which explains this finding [11,12]. *aggR *is a known diagnostic test target associated with EAEC virulence plasmids, which has shown better sensitivity than CVD432 in some studies, but is less specific [18-20].

**Table 1:** Genetic loci identified by IS3 profiling

**Band ref no**.	Band size	Criteria for selection	Sequence showing > 90% similarity at the nucleotide level (Genbank Accession #)	Subsequent evaluation of locus
650	1.7 kb	Present in several (13/22) EAEC, absent in K12	*E. coli *2-acylglycerophosphoethanolamine acyltransferase/acyl-acyl carrier protein synthetase gene (gi|290402)	Gene is present in the K-12 genome

651	1.2 kb	Present in ST31 and ST394 EAEC strains. Absent in all others.	*orfz2240 *(unknown function) from "O-island #62" of *E. coli *O157:H7 EDL933 genome (gi|12515207|gb|AE005358.1)	Present in ST31, ST38 and ST394-complex EAEC strains and EHEC O157. absent in other EAEC

652	0.35 kb	Present in K12 and EDL933, absent in *Shigella *and 16/22 EAEC. No phylogenetic association.	*E. coli racC *and *recE *genes, complete cds and 5' end (gi|147534|gb|M24905.1|ECORECEA)	Present in EAEC

708	0.65 kb	Absent in ST10 EAEC, present in most other strains	*E. coli *23 S rRNA gene, strain K12 DSM 30083T (gi|12053855|emb|AJ278710.1)	23 S rRNA, no diagnostic potential

711	0.5 kb	Present in 8/22 EAEC strains, and *Shigella*, absent in other strains	*E. coli aggR *gene for fimbrial adhesin activator (gi|471301|emb|Z32523.1|ECAGGRG)	Previously described plasmid-borne EAEC gene [17]

712	1.3 Kb	Absent in most EAEC (19/22), Present in K12	*E. coli *glycine decarboxylase (gi|NC 000913|U00096)	Gene is present in EAEC strains

The sequence derived from another band, predominant among the ST31 complex strains, also detected in the single ST394 strain, but absent in other EAEC, was 98% identical to *orfz2240 *from *E. coli *O157 strain EDL933 [21]. The z*2240 *open reading frame is located within the small (1,548 bp) O-island 62 of strain EDL933 and is also present in the genome of *E. coli *O157 Sakai (where it is annotated as Ecs2075 [22]) and four other O157:H7genomes. Similar loci (95% or greater identity over the entire sequence length) are present in the genomes of O55:H7 strain CB9615 (O55:H7 strains are believed to be the progenitors of O157 EHEC [23]), uropathogenic *E. coli *strains UMN026 and IAI39, multiresistant commensal SMS-3-5, as well as four *Shigella flexneri 2a *strains and a *Sh. sonnei *strain [24]. Like ST31 and ST394-complex EAEC, uropathogenic *E. coli *strains, and the single commensal, that have this island belong to ECOR group D [25]. O-island 62 is absent from all other 111 complete and 83 incomplete *E. coli *and related enterobacterial genomes that were publicly available by January 2011.

### Distribution of *orfz2240 *DNA among EAEC and non-EAEC

**Figure 2:**
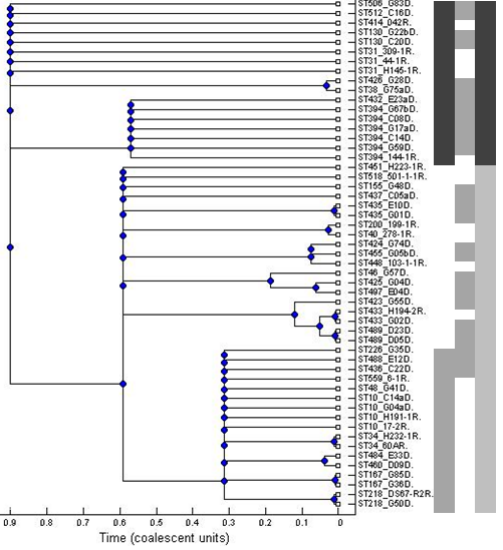
**Presence or absence *orfz2240 *mapped onto a 75% consensus ClonalFrame tree for MLST data from 53 EAEC strains including 46 strains from Nigerian children with diarrhoea (D) and cases of from other parts of the world (R)**. Principal *E. coli *sub-clades corresponding to three of the four major groups originally defined by MLEE - A, B1, and D - are marked in first the column to the immediate right of the tree respectively with light shading, no shading and dark shading. The central column indicates strain source with shaded strains from Nigeria and the far right column indicates presence (dark shading) or absence (light shading) of the *orfz2240 *locus.

Forty-six additional EAEC strains, not used in the profiling that initially identified *orfz2240*, were screened for *orfz2240 *by PCR, using primers 2240f and 2240r. These isolates were previously isolated from children with diarrhoea in an epidemiological study in Nigeria, and like the reference collection have been multilocus sequence-typed [12,26]. As shown in Figure 2, the *z2240 orf *was amplified from twelve of these strains. Two *z2240*-positive strains from Nigeria belonged to the ST complex 31 (STs130 and 512), seven to ST394, and two others belonged to the ST38 complex (STs 38 and 426), which shares *mdh *and *purA *alleles with ST31 and ST394 complexes and clusters with them by BURST and ClonalFrame analyses. The last strain (ST506) does not belong to a designated ST complex but is also an ECOR D EAEC strain [12,27]. Altogether (with the reference collection), this gene was detected in all 17 isolates from the ECOR D group sequence type complexes but was absent from the 51 isolates from all other sequence types including all isolates belonging to the most common EAEC ST complex, ST10.

We have previously found *chuA, fepC-*PAI and *lpf*-containing islands in EAEC strains belonging to ST31 and ST394 complexes [12,25]. These loci are also present in all ECOR group D EAEC and all three loci are present in EHEC O157 strains. Eighteen EHEC strains were screened for *orfz2240 *by hybridisation (Table 2). Only three isolates, all O157 strains, tested positive and all non-O157 EHEC strains lacked the gene. As also shown in Tables 2 and 3, *orfz2240 *was detected uncommonly outside the EHEC O157 and EAEC ECOR group D pathotypes. Important exceptions were diffusely-adherent *E. coli *and *Shigella sonnei*. Eight of eleven diffusely-adherent *E. coli *strains tested positive, as did 20 of 24 *Shigella sonnei *strains. We also screened 85 strains from 13 genera of enteric bacteria with probes for CVD432 and *orfz2240*. None of the isolates tested positive with the CVD432 probe and most were negative for *orfz2240*. Two *Aeromonas hydrophilia *gp isolates from diarrhoeal stools and none of four isolates of the same species from shellfish hybridised to the *z2240 *probe. Additionally, one of four *Morganella *spp., and one of six *Escherichia hermannii *strains hybridized to this probe (Table 2).

**Table 2:** Presence of *orfz2240 *in different pathogenic *E. coli *and other enteric bacteria

Diarrhoeagenic *E. coli *category or enterobacterial genus/species	# of strains screened	# positive for *z2240 *by PCR
EAEC (ST complexes 31,38 and 394)	16	16

EAEC (all other STs)	57	0

Enterohaemorrhagic *E. coli *O157:H7	3	3

Enterohaemorrhagic *E. coli *(non O157)	15	0

Diffusely adherent *E. coli*	11	8

*Shigella flexneri*	4	2

*Shigella dysenteriae*	3	0

*Shigella sonnei*	24	20

Enteroinvasive *E. coli*	4	0

Enterotoxigenic *E. coli*	2	0

Enteropathogenic *E. coli*	22	1

Uropathogenic *E. coli*	2	1

*Aeromonas hydrophilia *gp	6	2

*Citrobacter *sp.	14	0
*Escherichia hermanii*	6	1

*Enterobacter *sp.	7	0

*Hafnia *sp.	8	0

*Klebsiella *sp.	7	0

*Morganella *sp.	4	1

*Proteus *sp.	8	0

*Providencia *sp.	9	0

*Salmonella *sp.	6	0

*Serratia *sp.	4	0

*Vibrio *sp.	2	0

*Yersinia *sp.	4	0

**Table 3:** Properties of EAEC and DAEC strains used for IS3 profiling in this study

Strain	Serotype (where known)	Country of isolation	MLST-defined ST	ST complex	Adherence pattern	Virulence genes	CVD 432	*orf1600 (z2240)*
NA H191-1		Peru	10	10	Weak Diffuse	*pic*	-	-

AA 17-2	O3:H2	Chile	10	10	Aggregative-detaching	*aagA, aggR, aap*	+	-

AA 253-1	O3:H2	Thailand	10	10	Aggregative	*aagA, aggR, aap*	+	-

AA H232-1		Peru	34	10	Aggregative-detaching	*aggR, aap, pic*	+	-

AA 60A		Mexico	34	10	Aggregative	*aagA, aggR, aap, pic*	+	-

AA DS67-R2		Philippines	218	10	Aggregative	*aagA, aggR, aap*	+	-

AA 435-1	O33:H16	Thailand	295	10	Aggregative	*pet, aafA, aggR, aap, pic*	+	-

AA 103-1	O148:H28	Thailand	448	10	Aggregative	*Aap*	+	-

AA 501-1	OR:H53	Thailand	518	10	Aggregative	-	-	-

AA H194-2		Peru	433	10	Aggregative	*aagA, aggR, aap, pic*	+	-

AA 6-1	OR:H2	Thailand	559	10	Aggregative	*aggR, aap*	+	-

AA DS65-R2		Philippines	unknown	unknown	Weak localized-aggregative	-	-	-

AA H223-1		Peru	451	None	Aggregative	*aggR, aap*	+	-

AA H38-1		Peru	31	31	Aggregative	*aagA, aggR, aap*	+	+

AA 44-1	O36:H18	Thailand	31	31	Aggregative	*aggR, aap, pic*	+	+

AA H145-1		Peru	31	31	Aggregative	*aagA, aggR, aap, pic*	+	+

AA 309-1	O130:H27	Thailand	31	31	Aggregative	*aagA, aggR, aap, pic*	+	+

AA 042	O44:H18	Peru	414	31	Aggregative	*aafA, pet, aggR, aap, pic*	+	+

AA 144-1	O77:NM	Thailand	394	394	Aggregative-detaching	*aggR, aap*	+	+

AA 278-1	O125ac:H21	Thailand	40	155	Aggregative	*aggR, aap, pic*	+	-

AA 239-1	OR:H21	Thailand	40	155	Aggregative	*aggR, pic*	+	-

AA 199-1	OR:H1	Thailand	200	155	Aggregative	*pet, aafA, aggR, aap, pic*	+	-

Key: Genes encoded by: *aafA *= structural subunit of the aggregative adherence fimbriae I (AAF/I), *aap *= antiaggregative protein (dispersin), *aggA *= structural subunit of the aggregative adherence fimbriae II (AAF/II), *aggR *= aggregative adherence regulator, *pet*= plasmid-encoded enterotoxin, *pic*= mucinase,

### The EAEC equivalent of O-island 62 is similar but not identical to the EHEC island

**Figure 3:**
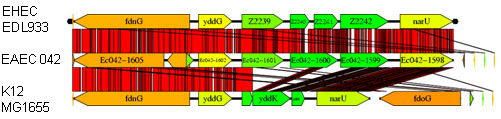
**EHEC O157:H7 strain EDL933 genome segment 2016573-2026572, containing O-island 62, and the corresponding regions in ECOR D EAEC strain 042 and *E. coli *K12 strain MG1655, illustrating the mosaic nature of the island**.

The flanking sequence of the cloned fragment, retrieved from the EAEC 042 genome, demonstrated that the EHEC O157 and EAEC 042 islands are of similar size and sequence, being 95% identical at the nucleotide level, but there are important differences in their predicted proteins (Figure 3). O-island 62 of EHEC strain EDL933 (and the equivalent and virtually identical island from EHEC O157 Sakai) is between the K-12 open reading frames *yddG *and *narU*. It is comprised of four open reading frames, annotated *z2239-z2242*. By contrast, the 042 island contains three open reading frames, *orfs 1601-1599*, the middle orf, *orf1600*, is a concatenate of EDL933 orfs *z2240 *and *z2241 *(Figure 3). A frameshift at position 70-71 (with respect to the 042 *orf1600 *sequence), results in a premature stop codon in *z2240 *of EHEC. The two predicted EHEC orfs thus generated show very high similarity to the 5' and 3' ends of the EAEC open reading frame (92% and 94% identical at the amino acid level respectively). Other O157 strains also have the EDL933 variety of the island.

In place of these genes, *E. coli *K-12 strain MG1655 carries three predicted open-reading frames *yddL, yddK *and *yddJ*. Predicted open reading frames *yddL *and *yddJ *are very small, with significant similarity to the 5' end of EHEC strain EDL933 orf *z2239 *and the 3' end of *z2242 *respectively (Figure 2). Therefore, although the entire island was probably acquired relatively recently in evolutionary time (its GC content, depending on strain, ranges between 33 and 36% compared to 48-50% for flanking DNA), it is likely that the EHEC or EAEC varieties represent the ancestral island, and that this was disrupted in *E. coli *K-12 by insertion of *yddK*. YddK is another predicted leucine-rich repeat protein and possible glycoprotein, with a predicted RNAse inhibitor domain found in most *E. coli *genomes and essential to *E. coli *K-12 [28].

## Discussion

Pathogen genomes contain genomic islands that are absent in non-pathogens. At least some of these islands contribute to virulence. Genomic islands may have been acquired by the common ancestor of a pathogenic lineage in which case they can serve as a marker for the lineage irrespective of their present contribution to virulence. Although some genomic islands have been described, much less is known about chromosomal EAEC virulence loci than plasmid-borne genes. Recent ordering of EAEC lineages by MLST has allowed us to conduct a within- and between-lineage search for unique DNA. The objective of this study was to identify conserved genetic loci among principal EAEC lineages. We hypothesised that EAEC strains, or subgroups of them would harbour conserved chromosomal loci and that identifying them would serve to improve the understanding of these pathogens, enhance their identification for research and clinical purposes and potentially find vaccine candidates.

Identification of factors that are common to pathogenic bacteria but absent in non-pathogens is an approach that has been shown to have promise for identifying virulence loci and candidate antimicrobial targets. For example, [29] used *in silico *methods to mine sequenced genomes for pathogen-specific factors. As there is only one completed EAEC genome, and just three others are in progress, we elected to use lower-resolution PCR-based genetic profiling to compare 22 genomes. Since a number of genomic islands contain, or are proximal to IS3 elements, we hypothesised that IS3-based profiling would identify loci that are lineage specific, and which might contribute to virulence. Using this approach, we were able to identify two diagnostic candidates, *aggR *and *orf1600*. The former is a transcriptional activator that has been characterised functionally and used to detect EAEC in epidemiological surveys [17,19]. The second target we identified is within an island present in EHEC O157 strains (as *orfz2240*) and in EAEC strains (*orf1600*) belonging to the ECOR D lineage. Compared to *in silico *methods, our approach yielded few hits. However, the small size of the *z2240/orf1600 *island and the *aggR *gene mean that the loci identified by IS3 profiling could be overlooked by other approaches.

The functionally-characterised protein showing greatest similarity to the predicted product of EHEC *orfz2240*/EAEC *orf1600 *is the invasion plasmid antigen H (IpaH) of *Shigella*. Amino acid residues 4-60 of Z2240 (and of EAEC Orf1600) are 35.8% identical to residues 3-119 of the 532 amino-acid IpaH variant (accession number gi152747). Each *Shigella *strain has multiple variants of IpaH which are more similar to each other than to Z2240, and vary in length. IpaH is an E3 ubiquitin ligase and is temporally associated with *Shigella *pathogenicity [30-32] Z2241 is predicted to be a leucine-rich protein of unknown function. If it is expressed, the EAEC hybrid Orf1600 could represent a bifunctional protein. However, EAEC strains appear to be mucosal pathogens and therefore it is not clear if a ubiquitin ligase, which might have a role in targeting intracellular proteins to the proteosome, would contribute to pathogenicity in this pathotype. Multiple attempts to over-express EAEC *orf1600 *for purification (data not shown) were unsuccessful, most likely due to toxicity. This, with comparative analysis of *E. coli *genomes, suggests that the 042 version of the island, and *orf1600 *in particular, may be under negative selection.

It is not known whether any or all of the versions of this island make functional proteins but this does not preclude expression or functional data emerging from future studies. However, identification of two targets, one previously unreported, offers proof-of-principle of our method for identifying general and lineage-specific EAEC loci. Following the realisation that the EAEC category is comprised of multiple pathotypes, convenient markers for significant lineages are needed to help determine their epidemiological significance. One such lineage is ECOR phylogenetic group D EAEC, which is globally disseminated and includes prototypical EAEC strain 042 that produced diarrhoea in three of five volunteers during a human challenge experiment [3]. The EAEC ECOR group D lineage contains strains belonging to ST31-, ST394- and ST38-complexes. ST394-complex EAEC were isolated much more frequently from Nigerian children with diarrhoea than from controls and after ST10, this complex was the most common in that population [12,25]. All the ST394-complex isolates in the *E. coli *MLST database appear to be EAEC strains and therefore this ST-complex represents a common complex that is very likely EAEC-specific. ST38 was much less frequently isolated from Nigerian children but was the only complex detected more than three times that was not recovered from controls, suggesting that it may represent a truly virulent lineage [12]. The island reported here could serve as a marker for the EAEC ECOR D lineage and combining the 2240 probe with commonly-employed diagnostic probes that detect the plasmid marked by CVD432, could help to determine the specific contribution of these EAEC pathotypes to the burden of diarrhoeal disease.

## Conclusion

A genomic island 95% identical to EHEC O157 O-island 62 is present in EAEC strains belonging to the ECOR D lineage. An open reading frame on this island, annotated as *orf1600 *in the EAEC 042 genome, can be used to identify this important EAEC lineage and the IS3 profiling method used to identify this locus can be used to identify conserved DNA in important enterobacterial lineages.

## Materials and methods

### Bacterial Strains

Twenty-two enteroaggregative *E. coli *strains from diverse geographical locations that have recently been typed by mutilocus sequence typing (MLST) constituted a reference collection of EAEC strains (Table 3) [12]. The collection was comprised of strains belonging to EAEC sequence types (STs) that are globally disseminated, most prominently ST10 and ST31 complexes and included two ST complexes (ST10 and ST394) that are predominantly recovered from individuals with diarrhoea [12]. Non-EAEC *E. coli *strains that were used as negative controls were *E. coli *K-12 strain MG1655, enterohaemorrhagic *E. coli *(EHEC) strain EDL933 (ATCC 43895) [33], diffusely adherent *E. coli *strains DA WC212-11 and DA H92-1, enteropathogenic *E. coli *strains E2348/69 and B171-8 [34,35], uropathogenic *E. coli *strain 536 [36], as well as *Shigella flexneri *2a strain 2457T [37]. *E. coli *K-12 strain DH5α (Sambrook and Russell, 2001) was used as the host strain for clones.

Forty-six EAEC strains previously isolated from children with diarrhoea in Nigeria [26] as well as 90 other non-EAEC isolates belonging to the enteropathogenic, enterohaemorrhagic, enterotoxigenic, enteroinvasive/*Shigella*, diffusely adherent and uropathogenic *E. coli *categories, plus 85 isolates from related genera, were employed to determine the distribution of loci found in this study [18,26,38]. Strains were maintained by cryopreservation in Luria Bertani Broth (LB) with 15% v/v glycerol at -70°C.

### Routine molecular biology procedures

Standard molecular biology procedures were employed [39]. Unless otherwise stated, DNA amplifications were performed using 1 unit recombinant *Taq *polymerase enzyme, 2 mM MgCl_2_, PCR buffer (Invitrogen) and 1 μM oligonucleotide primer in each reaction. All amplifications began with a two minute hot start at 94°C followed by 30 cycles of denaturing at 94°C for 30 s, annealing for 30 s at 5°C below primer annealing temperature and extending at 72°C for 1 minute for every Kb of DNA. PCR reactions were templated with genomic DNA or boiled bacterial colonies. Where necessary, *Taq *polymerase amplified products were TA-cloned into the pGEM-T vector (Promega) according to manufacturer's recommendations. They were then transformed into chemically competent *E. coli *K-12 DH5α cells and selected on plates containing ampicillin (100 μg/ml). Clones were verified by plasmid purification, restriction analysis and sequencing.

### IS3-based PCR profiling

Insertion element 3 (IS3)-based PCR profiling was performed using the IS3A primer (5'-CACTTAGCCGCGTGTCC-3') in the method described by Thompson et al. [16]. Use of this primer alone in this low-stringency protocol [16], rather than in conjunction with IS3B, gave profiles of suitable discriminatory strength, band intensity and resolution for evaluation and excision. Twenty-two EAEC reference strains belonging to 15 STs and including one untyped strain, plus two diffusely-adherent *E. coli *and three *E. coli *strains for which published genomic sequence is available were profiled. A 25 μl IS3 PCR reaction mixture was prepared for each isolate in a 0.5 ml thin-walled tube, using 200 ng (2 μl) of DNA and 23 μl of a PCR master mixture containing 10 mM Tris-HCl (pH 8.3), 50 mM KCl (1× PCR buffer, Invitrogen), a 400 μM concentration of each of dATP, dCTP, dGTP, and dTTP, 3 mM MgCl_2_, 1 unit of *Taq *DNA polymerase (Invitrogen), and primer IS3A at 6 μM. The amplification program consisted of an initial denaturation at 94°C for 5 min; 50 cycles of 94°C for 1 min, 35°C for 1 min, and 72°C for 2 min; and a final 7-min extension at 72°C. The amplification products were resolved by electrophoresis in 1.5% (w/v) agarose gels [20 cm (W) × 25 cm (L)] and were detected by ethidium bromide staining. For control purposes, the selected strains were compared to non-EAEC strains. Bands that were reproducibly common to several EAEC, but absent in non-EAEC controls were cloned and sequenced. Bands present in the controls but absent in EAEC were also sought. Other bands were selected because they were present or absent in specific EAEC phylogenetic groups. Bands of interest were excised and extracted using the QIAquick gel extraction kit (Qiagen), cloned into the TA vector pGEM-T and sequenced.

### Sequence analyses

FASTA-formatted sequences, with vector sequence removed, were analysed by BLAST-N (nucleotide-nucleotide Basic Local Alignment Search Tool at http://www.ncbi.nlm.nih.gov/BLAST[40]). Flanking genetic sequence was retrieved from coliBASE at http://xbase.bham.ac.uk/colibase/ and genomic islands were also mapped and compared at this site using the integrated Artemis and Artemis Comparison Tool [41,42].

Phylogenetic inferences about ancestral allelic MLST profiles and strain interrelatedness were made using eBURST version 3 http://eburst.mlst.net/ and ClonalFrame version 1.1 http://www.xavierdidelot.xtreemhost.com/clonalframe.htm[43,44]. Clonal complexes were defined using eBURST based on groups sharing six identical alleles and bootstrapping with 1000 samplings. Relationships among different sequence type complexes were inferred using ClonalFrame [44], a Bayesian method of constructing evolutionary histories that takes both mutation and recombination into account. For each analysis, four independent runs of the Markov chain were employed. ClonalFrame was used to compare independent runs by the method of Gelman and Rubin [45]. Calculated Gelman-Rubin statistics for all parameters were below 1.20, indicating satisfactory convergence between tree replicates. A 75% consensus tree was created for the EAEC isolates.

### DNA hybridisation

The EDL933 *orfz2240 *equivalent (part of *orf1600*) was amplified from EAEC strain 042 using primers 2240f (5'-CCATCTCCAGCAATTTTTGTG-3') and 2240r (5'-GCGCTTCCAGATTAACCATGAA-3'). The resulting 545 bp product was cloned into pGEM-T to produce plasmid pLRM3. The 2240 DNA probe was excised from pLRM3 with the enzymes *Pst*I and *Eco*RI. The fragment probe was gel purified using a Qiagen agarose gel extraction kit, then labelled with digoxigenin-11-dUTP using a random prime labelling kit (Roche Diagnostics). Labelled DNA probe was used in colony hybridisation reactions as described previously [46]. Briefly, test and control strains were inoculated into brain heart infusion broth and incubated in an orbital shaker (150 rpm) incubator for 16-18 hours at 37°C. Broth cultures were then inoculated onto nylon membranes (Hybond-N, Amersham) on the surface of brain heart infusion agar and incubated for 4-6 hours at 37°C. Colonies were lysed and the DNA was bound to the membrane by sequential treatment with sodium hydroxide/SDS, Tris-HCl/EDTA, saline sodium citrate solution and exposure of the membrane to ultraviolet light [39]. Bound target DNA was detected by hybridisation with the digoxigenin-labelled DNA probe followed by detection of the digoxigenin label by a monoclonal phosphatise-conjugated secondary antibody and a colour substrate for the enzyme. Reagents for immunological detection were supplied by Roche Diagnostics and detection of labelled DNA was performed in accordance with their instructions.

## Abbreviations

EAEC: enteroaggregative *Escherichia coli*; EHEC: enterohemorrhagic *Escherichia coli*; IS-3: insertion sequence 3; MLST: mult-ilocus sequence typing; ST: sequence type.

## Competing interests

The authors declare that they have no competing interests.

## Authors' contributions

INO conceived the study performed the IS-3 profiling, identified the hits, performed computational analyses and drafted the manuscript. LMS cloned the diagnostic probe and performed most of the validation. JNF contributed to validation. AMS contributed to validation, coordinated the project and helped to draft the manuscript. All authors read and approved the final manuscript.

## References

[B1] Huang DB Okhuysen PC Jiang ZD DuPont HL Enteroaggregative *Escherichia coli*: an emerging enteric pathogen Am J Gastroenterol 2004 99 383 389 10.1111/j.1572-0241.2004.04041.x 15046233

[B2] Mathewson JJ Johnson PC DuPont HL Pathogenicity of enteroadherent *Escherichia coli *in adult volunteers J Infect Dis 1986 154 524 527 10.1093/infdis/154.3.524 3525699

[B3] Nataro JP Deng Y Cookson S Cravioto A Savarino SJ Guers LD Levine MM Tacket CO Heterogeneity of enteroaggregative *Escherichia coli *virulence demonstrated in volunteers J Infect Dis 1995 171 465 468 10.1093/infdis/171.2.465 7844392

[B4] Cobeljic M Miljkovic-Selimovic B Paunovic-Todosijevic D Velickovic Z Lepšanovic Z Zec N Savic D Ilic R Konstantinovic S Jovanovic B Kostic V Enteroaggregative *Escherichia coli *associated with an outbreak of diarrhoea in a neonatal nursery ward Epidemiol Infect 1996 117 11 16 10.1017/S0950268800001072 8760945 PMC2271665

[B5] Itoh Y Nagano I Kunishima M Ezaki T Laboratory investigation of enteroaggregative *Escherichia coli *O untypeable:H10 associated with a massive outbreak of gastrointestinal illness J Clin Microbiol 1997 35 2546 2550 9316905 10.1128/jcm.35.10.2546-2550.1997 PMC230008

[B6] Jiang ZD Okhuysen PC Guo DC He R King TM DuPont HL Milewicz DM Genetic Susceptibility to enteroaggregative *Escherichia coli *diarrhea: polymorphism in the interleukin-8 promotor region J Infect Dis 2003 188 506 511 10.1086/377102 12898436

[B7] DuPont HL What's new in enteric infectious diseases at home and abroad Curr Opin Infect Dis 2005 18 407 412 10.1097/01.qco.0000182535.54081.68 16148527

[B8] Baudry B Savarino SJ Vial P Kaper JB Levine MM A sensitive and specific DNA probe to identify enteroaggregative *Escherichia coli*, a recently discovered diarrheal pathogen J Infect Dis 1990 161 1249 1251 10.1093/infdis/161.6.1249 2189007

[B9] Sheikh J Czeczulin JR Harrington S Hicks S Henderson IR Le Bouguenec C Gounon P Phillips A Nataro JP A novel dispersin protein in enteroaggregative *Escherichia coli* J Clin Invest 2002 110 1329 1337 12417572 10.1172/JCI16172 PMC151617

[B10] Okeke IN Nataro JP Enteroaggregative *Escherichia coli* Lancet Infect Dis 2001 1 304 313 10.1016/S1473-3099(01)00144-X 11871803

[B11] Johnson TJ Nolan LK Pathogenomics of the virulence plasmids of *Escherichia coli* Microbiol Mol Biol Rev 2009 73 750 774 10.1128/MMBR.00015-09 19946140 PMC2786578

[B12] Okeke IN Wallace-Gadsden F Simons H Matthews N Labar A Hwang J Wain J The enteroaggregative *Escherichia coli *category is comprised of multiple pathotypes from diverse lineages PLoS One 2010 5 e14093 10.1371/journal.pone.0014093 21124856 PMC2990770

[B13] Chaudhuri RR Sebaihia M Hobman JL Webber MA Leyton DL Goldberg MD Cunningham AF Scott-Tucker A Ferguson PR Thomas CM Complete genome sequence and comparative metabolic profiling of the prototypical enteroaggregative *Escherichia coli *strain 042 PLoS ONE 2010 5 e8801 10.1371/journal.pone.0008801 20098708 PMC2808357

[B14] Sawyer SA Dykhuizen DE DuBose RF Green L Mutangadura-Mhlanga T Wolczyk DF Hartl DL Distribution and abundance of insertion sequences among natural isolates of *Escherichia coli* Genetics 1987 115 51 63 3030884 10.1093/genetics/115.1.51 PMC1203063

[B15] Birkenbihl RP Vielmetter W Complete maps of IS1, IS2, IS3, IS4, IS5, IS30 and IS150 locations in *Escherichia coli *K12 Mol Gen Genet 1989 220 147 153 10.1007/BF00260869 2558284

[B16] Thompson CJ Daly C Barrett TJ Getchell JP Gilchrist MJ Loeffelholz MJ Insertion element IS3-based PCR method for subtyping *Escherichia coli *O157:H7 J Clin Microbiol 1998 36 1180 1184 9574672 10.1128/jcm.36.5.1180-1184.1998 PMC104795

[B17] Nataro JP Yikang D Yingkang D Walker K AggR, a transcriptional activator of aggregative adherence fimbria I expression in enteroaggregative *Escherichia coli* J Bacteriol 1994 176 4691 4699 7913930 10.1128/jb.176.15.4691-4699.1994 PMC196291

[B18] Okeke IN Lamikanra A Czeczulin J Dubovsky F Kaper JB Nataro JB Heterogeneous virulence of enteroaggregative *Escherchia coli *strains isolated from children in Southwest Nigeria J Infect Dis 2000 181 252 260 10.1086/315204 10608774

[B19] Cerna JF Nataro JP Estrada-Garcia T Multiplex PCR for detection of three plasmid-borne genes of enteroaggregative *Escherichia coli *strains J Clin Microbiol 2003 41 2138 2140 10.1128/JCM.41.5.2138-2140.2003 12734261 PMC154749

[B20] Yamazaki M Inuzuka K Matsui H Sakae K Suzuki Y Miyazaki Y Ito K Plasmid encoded enterotoxin (Pet) gene in enteroaggregative *Escherichia coli *isolated from sporadic diarrhea cases Jpn J Infect Dis 2000 53 248 249 11227025

[B21] Perna NT Plunkett G Burland V Mau B Glasner JD Rose DJ Mayhew GF Evans PS Gregor J Kirkpatrick HA Genome sequence of enterohaemorrhagic *Escherichia coli *O157:H7 Nature 2001 409 529 533 10.1038/35054089 11206551

[B22] Hayashi T Makino K Ohnishi M Kurokawa K Ishii K Yokoyama K Han CG Ohtsubo E Nakayama K Murata T Complete genome sequence of enterohemorrhagic *Escherichia coli *O157:H7 and genomic comparison with a laboratory strain K-12 DNA Res 2001 8 11 22 10.1093/dnares/8.1.11 11258796

[B23] Reid S Herbelin C Bumbaugh A Selander R Whittam T Parallel evolution of virulence in pathogenic *Escherichia coli* Nature 2000 406 64 67 10.1038/35017546 10894541

[B24] Wei J Goldberg MB Burland V Venkatesan MM Deng W Fournier G Mayhew GF Plunkett G Rose DJ Darling A Complete genome sequence and comparative genomics of *Shigella flexneri *serotype 2a strain 2457T Infect Immun 2003 71 2775 2786 10.1128/IAI.71.5.2775-2786.2003 12704152 PMC153260

[B25] Wallace-Gadsden F Wain J Johnson JR Okeke IN Enteroaggregative *Escherichia coli *related to uropathogenic Clonal Group A Emerg Infect Dis 2007 13 757 760 17553259 10.3201/eid1305.061057 PMC2738470

[B26] Okeke IN Lamikanra A Steinruck H Kaper JB Characterization of *Escherichia coli *strains from cases of childhood diarrhea in provincial southwestern Nigeria J Clin Microbiol 2000 38 7 12 10618054 10.1128/jcm.38.1.7-12.2000 PMC86005

[B27] Wirth T Falush D Lan R Colles F Mensa P Wieler LH Karch H Reeves PR Maiden MC Ochman H Achtman M Sex and virulence in *Escherichia coli: *an evolutionary perspective Mol Microbiol 2006 60 1136 1151 10.1111/j.1365-2958.2006.05172.x 16689791 PMC1557465

[B28] Winterberg KM Luecke J Bruegl AS Reznikoff WS Phenotypic screening of *Escherichia coli *K-12 Tn5 insertion libraries, using whole-genome oligonucleotide microarrays Appl Environ Microbiol 2005 71 451 459 10.1128/AEM.71.1.451-459.2005 15640221 PMC544249

[B29] Stubben C Duffield M Cooper I Ford D Gans J Karlyshev A Lingard B Oyston P de Rochefort A Song J Steps toward broad-spectrum therapeutics: discovering virulence-associated genes present in diverse human pathogens BMC Genomics 2009 10 501 10.1186/1471-2164-10-501 19874620 PMC2774872

[B30] Toyotome T Suzuki T Kuwae A Nonaka T Fukuda H Imajoh-Ohmi S Toyofuku T Hori M Sasakawa C *Shigella *protein IpaH(9.8) is secreted from bacteria within mammalian cells and transported to the nucleus J Biol Chem 2001 276 32071 32079 10.1074/jbc.M101882200 11418613

[B31] Zhu Y Li H Hu L Wang J Zhou Y Pang Z Liu L Shao F Structure of a *Shigella *effector reveals a new class of ubiquitin ligases Nat Struct Mol Biol 2008 15 1302 1308 10.1038/nsmb.1517 18997779

[B32] Singer AU Rohde JR Lam R Skarina T Kagan O Dileo R Chirgadze NY Cuff ME Joachimiak A Tyers M Structure of the *Shigella *T3SS effector IpaH defines a new class of E3 ubiquitin ligases Nat Struct Mol Biol 2008 15 1293 1301 10.1038/nsmb.1511 18997778 PMC2764551

[B33] Riley L Remis R Helgerson S McGee H Wells J Davis B Hebert R Olcott E Johnson L Hargrett N Hemorrhagic colitis associated with a rare *Escherichia coli *serotype N Engl J Med 1983 308 681 685 10.1056/NEJM198303243081203 6338386

[B34] Paulozzi LJ Johnson KE Kamahele LM Clausen CR Riley LW Helgerson SD Diarrhea associated with adherent enteropathogenic *Escherichia coli *in an infant and toddler center, Seattle, Washington Pediatrics 1986 77 296 300 3513114

[B35] Levine MM Nataro JP Karch H Baldini MM Kaper JB Black RE Clements ML O'Brien A The diarrheal response of humans to some classic serotypes of enteropathogenic *Escherichia coli *is dependent on a plasmid encoding an enteroadhesiveness factor J Infect Dis 1985 152 550 559 10.1093/infdis/152.3.550 2863318

[B36] Blum G Ott G Lischewski A Ritter A Imrich H Tschäpe H Hacker J Excision of large DNA regions termed pathogenicity islands from the tRNA-specific loci in the chromosome of an *Escherichia coli *wild-type pathogen Infect Immun 1994 62 606 614 7507897 10.1128/iai.62.2.606-614.1994 PMC186147

[B37] Du Pont HL Hornick RB Dawkins AT Snyder MJ Formal SB The response of man to virulent *Shigella flexneri*2a J Infect Dis 1969 119 296 299 10.1093/infdis/119.3.296 5780532

[B38] Okeke IN Borneman JA Shin S Mellies JL Quinn LE Kaper JB Comparative sequence analysis of the plasmid-encoded regulator of enteropathogenic *Escherichia coli *strains Infect Immun 2001 69 5553 5564 10.1128/IAI.69.9.5553-5564.2001 11500429 PMC98669

[B39] Sambrook J Russell DW Molecular cloning: a laboratory manual 2001 3 Cold Spring Harbor, N.Y.: Cold Spring Harbor Laboratory Press

[B40] Altschul SF Gish W Miller W Myers EW Lipman DJ Basic local alignment search tool J Mol Biol 1990 215 403 410 2231712 10.1016/S0022-2836(05)80360-2

[B41] Rutherford K Parkhill J Crook J Horsnell T Rice P Rajandream MA Barrell B Artemis: sequence visualization and annotation Bioinformatics 2000 16 944 945 10.1093/bioinformatics/16.10.944 11120685

[B42] Carver TJ Rutherford KM Berriman M Rajandream MA Barrell BG Parkhill J ACT: the Artemis Comparison Tool Bioinformatics 2005 21 3422 3423 10.1093/bioinformatics/bti553 15976072

[B43] Feil EJ Li BC Aanensen DM Hanage WP Spratt BG eBURST: inferring patterns of evolutionary descent among clusters of related bacterial genotypes from multilocus sequence typing data J Bacteriol 2004 186 1518 1530 10.1128/JB.186.5.1518-1530.2004 14973027 PMC344416

[B44] Didelot X Falush D Inference of bacterial microevolution using multilocus sequence data Genetics 2007 175 1251 1266 10.1534/genetics.106.063305 17151252 PMC1840087

[B45] Gelman A Rubin D Inference from iterative simulation using multiple sequences Stat Sci 1992 7 457 511 10.1214/ss/1177011136

[B46] Chapman PA Daly CM Comparison of Y1 mouse adrenal cell and coagglutination assays for detection of *Escherichia coli *heat labile enterotoxin J Clin Pathol 1989 42 755 758 10.1136/jcp.42.7.755 2668342 PMC1142029

